# Mendelian randomisation and mediation analysis of self-reported walking pace and coronary artery disease

**DOI:** 10.1038/s41598-024-60398-8

**Published:** 2024-05-01

**Authors:** Iain R. Timmins, Francesco Zaccardi, Thomas Yates, Frank Dudbridge

**Affiliations:** 1 Department of Population Health Sciences, University of Leicester, Leicester, UK; 2 Division of Genetics and Epidemiology, Institute of Cancer Research, London, UK; 3 Statistical Innovation, Oncology R&D, AstraZeneca, Cambridge, UK; 4 Diabetes Research Centre, University of Leicester, Leicester, UK; 5 NIHR Leicester Biomedical Research Centre, University of Leicester and University Hospitals of Leicester NHS Trust, Leicester, UK

**Keywords:** Genome-wide association studies, Cardiovascular diseases, Cardiology, Risk factors

## Abstract

The aim of this study was to assess the causal relationship between habitual walking pace and cardiovascular disease risk using a Mendelian randomisation approach. We performed both one- and two-sample Mendelian randomisation analyses in a sample of 340,000 European ancestry participants from UK Biobank, applying a range of sensitivity analyses to assess pleiotropy and reverse causality. We used a latent variable framework throughout to model walking pace as a continuous exposure, despite being measured in discrete categories, which provided more robust and interpretable causal effect estimates. Using one-sample Mendelian randomisation, we estimated that a 1 mph (i.e., 1.6 kph) increase in self-reported habitual walking pace corresponds to a 63% (hazard ratio (HR) = 0.37, 95% confidence interval (CI), 0.25–0.55, *P* = 2.0 × 10^–6^) reduction in coronary artery disease risk. Using conditional analyses, we also estimated that the proportion of the total effect on coronary artery disease mediated through BMI was 45% (95% CI 16–70%). We further validated findings from UK Biobank using two-sample Mendelian randomisation with outcome data from the CARDIoGRAMplusC4D consortium. Our findings suggest that interventions that seek to encourage individuals to walk more briskly should lead to protective effects on cardiovascular disease risk.

## Introduction

Cardiovascular disease is a leading cause of morbidity and mortality worldwide^1^, where it is estimated that physical inactivity contributes significantly to the total disease burden^2^. Walking is of particular interest as a simple and accessible form of exercise^3^, with intervention studies suggesting a protective effect of increased walking activity on a broad range of cardiometabolic risk factors^4^. Moreover, while popular health goals typically focus on the amount of time spent walking^5^ or achieving a total number of steps per day^6^, more work is needed on the potential health benefits of habitual walking at a brisk pace. Additionally, previous research has shown walking pace to be a powerful predictor of survival^7,8^ and health outcomes, yet less is known about walking pace as a potential target for intervention.

Previous large-scale epidemiological studies have consistently shown that usual walking pace, typically self-reported through questionnaire, has a strong inverse association with cardiovascular disease risk^9–12^. Manson et al.^9^ examined the association between walking pace and coronary artery disease in the Nurses’ Health Study, a cohort of 72,488 female nurses who were 40–65 years old, observing that women who walked at a brisk pace (≥ 3 mph) compared to a slow pace (< 2 mph) had a 36% reduction in risk. Tanasescu et al.^10^ analysed a cohort of 44,452 men aged 40 through 75 years in the Health Professionals’ Follow-up Study (HPFS), observing that walking at a brisk pace (≥ 4 mph), compared with an easy pace (≤ 2 mph), was associated with reduction in risk of coronary artery disease of 49%. Additionally, a comprehensive meta-analysis by Hamer et al.^13^ incorporating data on 459,833 participants from 18 prospective cohort studies, identified a 48% reduction in cardiovascular disease risk when comparing the highest versus lowest categories of walking pace. However, it is not clear the extent to which these observational studies are prone to bias from factors such as residual confounding and reverse causation.

To further understand the potential health benefits of increased walking pace on cardiovascular risk, we therefore performed Mendelian randomisation, which is an instrumental variable approach that uses genetic variation associated with the exposure to test for a causal effect on an outcome^14^. Using this approach enables us to potentially estimate the exposure-outcome relationship with less confounding bias than conventional observational approaches.

We applied Mendelian randomisation analysis in UK Biobank, where participants self-reported their walking pace as “slow”, “steady/average” or “brisk”. While this approach has been used previously for testing causal effects of walking pace on cardiovascular outcomes in UK Biobank^15,16^, those studies have interpreted effect sizes in terms of per-category increases in walking pace. This, however, is problematic since the magnitude of Mendelian randomisation estimates based on coarsened, categorical exposures is not well-defined due to violations of the exclusion restriction criterion^17^. In our previous analysis^18^, we identified a 56% lower risk of coronary artery disease per category increase in usual walking pace (i.e., from slow to steady/average, or from steady/average to brisk pace), while Chen et al.^15^ recently identified a 45%, 69% and 44% lower risk of atrial fibrillation, heart failure and stroke, respectively, per category increase in walking pace. To address this issue, we analysed data on walking pace and cardiovascular risk in UK Biobank, applying the latent variable framework developed recently by Tudball et al.^19^, which assumes a latent continuous measure underlying self-reported categories of walking pace (analogous to disease liability underlying a binary diagnosis measure). In our study, the effect sizes are estimated in terms of this unmeasured, latent measure of walking pace, providing a more robust and interpretable estimate of the causal effect of increased walking pace on cardiovascular disease risk.

## Methods

### Study participants

We used data from UK Biobank^20^, an ongoing prospective cohort study, which recruited more than 500,000 participants (55% women) between March 2006 and July 2010 from 22 centres throughout England, Wales and Scotland. At recruitment, participants were aged between 40 and 69 years and lived within 25 miles of a study recruitment centre. An initial sample of 502,599 individuals consented to join the study. In the one-sample Mendelian randomisation analysis we included 344,268 unrelated European ancestry individuals with complete genotype, confounder and outcome data (Supplementary Fig. 1).

### Self-reported walking pace

A touchscreen questionnaire was used to capture usual walking pace at baseline. Participants were asked to answer the following question: “How would you describe your usual walking pace: slow; steady/average; brisk; none of the above; prefer not to answer?” Further information was available to participants which clarified a slow pace as < 3 miles per hour (mph), a steady/average pace as 3–4 mph, and a brisk pace as > 4 mph. We excluded participants whose answers were “None of the above” (n = 848) or “Prefer not to answer” (n = 169). The low numbers of these exclusions suggest minimal impact of any informative missingness.

### Outcomes

We identified incident fatal and non-fatal CAD events using information on hospital admissions (Hospital Episode Statistics, HES) linked to UK Biobank, based on the International Classification of Diseases (ICD) diagnostic codes (ICD-9: 410–412; ICD-10: I21–I24, I25.2) or coronary artery bypass graft (CABG) and percutaneous transluminal coronary angioplasty (PTCA) procedure codes (OPCS-4: K40 to K46, K49, K50.1, or K75), either in the primary or secondary position in the hospital records. Date and cause of death were obtained with linkage of UK Biobank to NHS Digital in participants from England and Wales and to the NHS Central Register in participants from Scotland. Participants were followed-up between study entry (baseline visit) until the occurrence of the study outcome or latest available censoring date that was available for this specific UK Biobank project (February 28, 2021 for England and Scotland; March 31, 2016 for Wales).

### Covariates

Data were also captured for the following putative risk factors: age, sex, systolic blood pressure, low-density lipoprotein (LDL) cholesterol, smoking status (current, former, never), history of diabetes mellitus (type 1 or type 2), social deprivation (Townsend deprivation index, with a higher index indicating a greater degree of deprivation), educational attainment, and participation in strenuous physical activity. Self-reported strenuous physical activity was a binary variable defined as spending at least 2–3 days/week or more doing strenuous sports (defined as sports that make you sweat or breathe hard) or other exercises (e.g., swimming, cycling) (UK Biobank Data Field [DF] 991), for a duration of 15–30 min or greater (DF 1001). We also considered measures of overall health status and mobility. We used an overall health status indicator based on data for 81 cancer and 443 non-cancer illnesses ascertained through self-reported questionnaire (DF 20001, 20002)^21^. A mobility limitation variable was defined based on participants having self-reported longstanding illness or disability (DF 2188), chest pain at rest (DF 2335) or leg pain while walking (DF 3606). Hand grip strength was assessed through the use of a hydraulic hand dynamometer (Jamar J00105) while sitting (DF 46, 47). Additionally, cardiorespiratory fitness was assessed on a subset of 59,056 participants through submaximal bicycle tests, with participants’ maximum workload calculated using formulas based on age, sex, weight, height and resting heart rate^22^.

### Genetic data

The initial genotyping, imputation and quality control were conducted centrally by UK Biobank and have been described in detail elsewhere^23^. In summary, genotyping was performed on 488,377 individuals using the UK BiLEVE Axiom Array and the UK Biobank Axiom arrays, with imputation to the Haplotype Reference Consortium panel^24^ and the merged UK10K and 1000 Genomes phase 3 reference panels^25^, which have in total approximately 96 million variants. We removed individuals with outlying heterozygosity and missingness, and excluded individuals where genetically inferred gender did not match the submitted gender, as well as excluding samples with putative sex chromosome aneuploidy. We restricted the sample to individuals of European ancestry, where ancestry was defined by the $k$-means clustering of the first two principal components. We further removed related individuals from the total sample such that no pair were related to 3rd degree or above, corresponding to a KING kinship coefficient^26^ of < 0.044.

### Latent variable model

The latent variable approach defines a threshold model that assumes those with latent walking pace above certain levels will express a higher self-reported category of walking pace^27^. The latent variable for walking pace is itself unmeasured, and is envisaged to have a continuous, normal distribution. Moreover, it includes both genetic and environmental factors, as well as random variation that influences self-reported categories of walking pace. In particular, we observed self-reported walking pace as a coarsened exposure $D$ with 3 ordered categories, which we assume is characterised by a discretisation of an unobserved continuous latent exposure $L \sim N({\mu }_{L}, {\sigma }_{L}^{2})$, where $L$ is self-reported walking pace measured in terms of miles per hour (mph). We have:

$$D = \left\{ { \begin{array}{*{20}l} 0 \hfill & {{\text{if}}\quad L \le 3\;{\text{mph}}} \hfill \\ 1 \hfill & {{\text{if}}\quad 3\;{\text{mph}} < L \le 4\;{\text{mph}}} \hfill \\ 2 \hfill & {{\text{if}}\quad 4\;{\text{mph}} < L} \hfill \\ \end{array} } \right.$$

Moreover, $D$ takes the values 0, 1 and 2 with frequencies $\pi_{k}$ for the three ordered categories, which can be estimated from the sample population. Using the cumulative distribution function ${\Phi }$ of the standard normal distribution, we have ${\mathbb{P}}\left( {L \le 3\;{\text{mph}}} \right) = \pi_{0}$ and ${\mathbb{P}}\left( {L \le 4\;{\text{mph}}} \right) = \pi_{0} + \pi_{1}$, and so $\left( {3 - \mu_{L} } \right)/\sigma_{L} = {\Phi }^{ - 1} (\pi_{0} )$ and $\left( {4 - \mu_{L} } \right)/\sigma_{L} = {\Phi }^{ - 1} (\pi_{0} + \pi_{1} )$. Hence, the standard deviation of $L$ in miles per hour can be calculated as:

$$\sigma_{L} = \frac{{\left( {4 - 3} \right)}}{{{\Phi }^{ - 1} (\pi_{0} + \pi_{1} ) - {\Phi }^{ - 1} (\pi_{0} )}}$$

which we use to scale effect size estimates in terms of miles per hour.

### One-sample Mendelian randomisation

For one-sample Mendelian randomisation analysis we used a set of 65 independent variants for walking pace derived using a clumping algorithm applied to summary statistics from Timmins et al.^18^. SNPs were clumped using a significance threshold *P* < 5 × 10^–8^, based on an LD threshold $r$^2^ ≤ 0.001 and a distance > 10,000 kb, using a reference panel of 10,000 randomly sampled European ancestry individuals in UK Biobank. The strength of each instrument was measured using the $F$-statistic^28^ (Supplementary Table 1). Participant characteristics across deciles of genetically predicted walking pace based on these 65 SNPs are presented in Supplementary Table 2.

We used the one-sample Mendelian randomisation framework set out by Tudball et al. to estimate the effect of increases in latent self-reported walking pace on coronary artery disease risk^19^. First, we found the predicted genetic values of walking pace, defined as the linear predictor from an ordered probit regression of walking pace on the set of 65 genetic variants. Second, the linear predictor was standardised by its SD. Third, a Cox proportional hazards model was fitted of coronary artery disease status on the standardised linear predictor, with adjustment for age, sex, genotyping array and 20 principal components. Fourth, the resulting standardised effect estimate ${\beta }_{G}$ was scaled by the proportion of variance explained by the instruments on the latent scale, denoted by ${\theta }^{2}$, which is a sensitivity parameter and was varied across a plausible range of values. The resulting latent scale estimate ${\beta }_{L}={\beta }_{G}/(\theta {\sigma }_{L})$ could then be interpreted as the effect on coronary artery disease risk per 1 mph increase in self-reported walking pace. We computed ${\theta }^{2}$ by first estimating the proportion of phenotypic variance in walking pace explained by the 65 genetic variants on the observed scale (coded 0, 1 and 2 for slow, steady/average and brisk pace, respectively), which we then transformed to the latent scale^18^. Standard errors of effect estimates were derived by bootstrapping with 1000 resamples.

Additionally, as a sensitivity analysis, we applied MR Steiger filtering to understand whether the instruments had a direct influence on coronary artery disease or cardiovascular risk factors, rather than on walking pace^29^. Using the set of covariates within our UK Biobank analytical sample, we applied Steiger’s $Z$-test for correlated correlations^30^. We identified instruments with suggestive evidence of a direct influence on coronary artery disease or a cardiovascular risk factor, rather than on walking pace, at a suggestive threshold of *P*_Steiger_ < 0.05, and we repeated all the Mendelian randomisation analyses after removing these SNPs.

We tested the proportional hazards assumption by calculating the Pearson correlations between the scaled Schoenfeld residuals and rank-normalised natural logarithm of follow-up time^31^.

To facilitate comparison with the two-sample Mendelian randomisation, we also repeated the one-sample Mendelian randomisation analysis using logistic rather than Cox regression (Supplementary Table 3).

### Two-sample Mendelian randomisation

For validation we used outcome data external to UK Biobank, applying the two-sample framework described by Tudball et al.^19^. For coronary artery disease, we used publicly available genetic association estimates from the CARDIoGRAMplusC4D 1000 Genomes-based genome-wide association study meta-analysis of 60,801 cases and 123,504 controls^32^. A broad and inclusive cases status for coronary artery disease included acute coronary syndrome, myocardial infarction, angina with one angiographic stenosis of greater than 50%, and chronic stable angina. We matched 62 variants for walking pace with those available in the coronary artery disease GWAS summary statistics, using proxy SNPs with a minimum linkage disequilibrium (LD) of *r*^2^ = 0.8.

We estimated the effect size ${\widehat{\alpha }}_{j}$ of instruments on walking pace as coefficients from an ordered probit regression on our analytical sample of 344,268 individuals, adjusting for age, sex, genotyping array and 20 principal components, implemented in the Julia package *OrdinalGWAS.jl*^33^. Using the effect sizes ${\widehat{\alpha }}_{j}$ for walking pace, and the log-odds ratios for coronary artery disease for each instrument, we computed causal effect estimates by applying the inverse-variance weighted method^34^. Further analyses were performed using the weighted median^35^, robust adjusted profile score method^36^, and MR-Egger regression^37^ (oriented with SNPs positively associated with the walking pace exposure^38^), which adjust for inconsistency across the instruments, weak instrument bias, and unbalanced horizontal pleiotropy, respectively. For each causal effect estimate $\beta$, we standardised by the genetic share of the latent exposure by scaling our estimand to ${\beta }_{G}={\left(\sum {\widehat{\alpha }}_{j}^{2}{\sigma }_{{Z}_{j}}^{2}\right)}^{1/2}\beta$, where the variance ${\sigma }_{{Z}_{j}}^{2}$ for each instrument was calculated as 2${f}_{j}(1-{f}_{j})$ using the allele frequencies ${f}_{j}$, and the effect sizes ${\widehat{\alpha }}_{j}$ for walking pace were derived from the ordinal regression. Finally, we scaled again to find the latent scale estimate ${\beta }_{L}={\beta }_{G}/(\theta {\sigma }_{L})$, which is interpreted as the effect per 1 mph increase in self-reported walking pace.

Analyses were performed using the *MendelianRandomization* package implemented in R software^39^.

### Mediation analysis on BMI

We further investigated whether the effect of walking pace on coronary artery disease was mediated by BMI. Following the same steps above for the two-sample Mendelian randomisation approach, we performed a further analysis where we conditioned out the effect of BMI on both the exposure and outcome. For the SNP-exposure association, we estimated the effect size ${\widehat{\alpha }}_{j}$ of instruments on walking pace by adding BMI as a covariate in the ordered probit regression. For the SNP-outcome association, we adjusted the SNP effects on coronary artery disease (from CARDIoGRAMplusC4D summary statistics) for the effect of BMI using the mtCOJO^40^ approach, where we used summary statistics from a GWAS for BMI conducted by Neale Lab (nealelab.is/uk-biobank) which were derived from 361,194 individuals of white British ancestry from UK Biobank. Having conditioned out the effect of BMI in both the exposure and outcome, we performed univariable two-sample Mendelian randomisation using these conditional SNP effect sizes, which equates to estimating the direct effect of walking pace independent of BMI on coronary artery disease. We performed the same set of methods and sensitivity analyses described previously (MR-IVW, MR-WM, MR-RAPS and MR-EGGER). Having obtained the total effect, and now having estimated the direct effect, we could further identify the indirect effect and proportion mediated through BMI using the difference method^41^ on the log-odds scale. Standard errors for the indirect effect and proportion mediated were derived by parametric bootstrapping with 1000 resamples.

## Results

### Descriptive characteristics

In an analytical sample of 344,268 individuals, the median age at baseline was 58.3 years (interquartile range 50.7–63.3), and 187,863 (54.6%) were female. Over a median follow-up of 11.9 years (11.1–12.6 years), 9993 incident coronary artery disease events were observed.

The characteristics of the included cohort by walking pace are displayed in Table 1. The number of participants with slow (< 3 mph), steady/average (3–4 mph) and brisk (> 4 mph) walking pace was 24,454 (7.1%), 179,469 (52.1%) and 140,455 (40.8%), respectively. Brisk walkers were slightly younger, had lower BMI, lower systolic blood pressure, were less likely to be former or current smokers, and participated in more strenuous physical activity. Slower walkers had a higher deprivation index, fewer years of education, poorer fitness and overall health status, and a greater proportion had mobility limitations.

**Table 1:** Participant characteristics of analytical sample at baseline and by self-reported walking pace.

Variable	Slow	Steady/average	Brisk	Total sample
Participants	24,454 (7.1%)	179,369 (52.1%)	140,445 (40.8%)	344,268
Female gender	13,889 (56.8%)	98,723 (55.0%)	75,251 (53.6%)	187,863 (54.6%)
Age (years)	59.7 (7.3)	57.8 (7.9)	55.7 (8.0)	57.1 (8.0)
Body mass index (kg/m^2^)	31.3 (6.6)	27.9 (4.6)	25.8 (3.7)	27.3 (4.7)
Systolic blood pressure (mmHg)	140.2 (18.8)	139.1 (18.7)	136.1 (18.3)	138.0 (18.6)
LDL cholesterol (mmol/l)	3.4 (0.9)	3.6 (0.9)	3.6 (0.8)	3.6 (0.9)
Smoking status				
Never	10,477 (42.8%)	95,496 (53.2%)	81,523 (58.0%)	187,496 (54.5%)
Former	9644 (39.4%)	64,771 (36.1%)	46,899 (33.4%)	121,314 (35.2%)
Current	4333 (17.7%)	19,102 (10.6%)	12,023 (8.6%)	35,458 (10.3%)
Years of education	12.9 (5.3)	14.6 (5.1)	16.0 (4.7)	15.0 (5.1)
Townsend deprivation index	− 0.3 (3.4)	− 1.5 (3.0)	− 1.7 (2.8)	− 1.5 (3.0)
History of diabetes	3206 (13.1%)	8386 (4.7%)	3052 (2.2%)	14,644 (4.3%)
Participate in strenuous physical activity	2730 (11.2%)	44,323 (24.7%)	48,572 (34.6%)	95,625 (27.8%)
Health status (classified as healthy)	8910 (36.4%)	116,996 (65.2%)	101,276 (72.1%)	227,182 (66.0%)
Mobility limitation	19,652 (80.4%)	72,715 (40.5%)	44,485 (31.7%)	136,852 (39.8%)
Grip strength (kg)	25.8 (11.1)	30.4 (10.9)	32.4 (10.8)	30.9 (11.0)
Cardiorespiratory fitness* (mL kg^−1^ min^−1^)	31.1 (9.6)	34.7 (9.9)	38.3 (10.4)	36.2 (10.3)
Coronary artery disease events	1250 (5.1%)	5784 (3.2%)	2959 (2.1%)	9993 (2.9%)

Data shown as number and percentage for categorical variables, mean and standard deviation for continuous variable. *Assessed on a subset of 59,056 participants.

### One-sample Mendelian randomisation analysis

The strength of the genetic instruments denoted by the F-statistic was ≥ 10 for each walking pace SNP, varying between 10.8 and 70.7 (full details are presented in Supplementary Table 1). The 65 genetic variants explained 0.72% of genetic variance in self-reported walking pace on the latent scale, hence we varied ${\theta }^{2}$ between 0.0050, 0.0072 and 0.010. The standard deviation of self-reported walking pace on the latent scale was estimated as ${\sigma }_{L}$= 0.57 mph.

For ${\theta }^{2}$ = 0.0072, we estimated that a 1 mph increase in walking pace was associated with a 63% (HR = 0.37, 95% CI 0.25–0.55, *P* = 7.1 × 10^–7^) reduction in coronary artery disease risk. While similar magnitude associations were found when varying ${\theta }^{2}$ between 0.0050 and 0.010 (Table 2).

**Table 2:** One-sample Mendelian randomisation Cox regression analysis of self-reported walking pace on coronary artery disease in UK Biobank.

SNPs	θ^2^	Hazard ratio (95% CI) per 1 mph increase in walking pace	*P* value
66	0.0050	0.30 (0.19–0.48)	7.1 × 10^–7^
66	0.0072	0.37 (0.25–0.55)	7.1 × 10^–7^
66	0.0100	0.43 (0.30–0.60)	7.1 × 10^–7^
55 (Steiger-filtered)	0.0050	0.28 (0.17–0.47)	5.7 × 10^–7^
55 (Steiger-filtered)	0.0072	0.35 (0.23–0.53)	5.7 × 10^–7^
55 (Steiger-filtered)	0.0100	0.41 (0.29–0.58)	5.7 × 10^–7^

Effect per 1 mph increase in self-reported walking pace.

Using MR-Steiger filtering, we identified 11 SNPs (*P*_Steiger_ < 0.05) which had evidence of a stronger direct effect on coronary artery disease or related cardiovascular risk factors, or on overall health status and mobility, than on walking pace (Supplementary Table 4). Of these, 10 displayed evidence of a direct effect on BMI; in addition, 1 SNP (rs143384) showed evidence of a direct effect on hand grip strength. Notably, rs9972653 from the *FTO* locus had a far stronger direct effect on BMI than on walking pace (*P*_Steiger_ = 1.1 × 10^–56^). After filtering these 11 SNPs, we observed no material change in the strength of associations. In particular, for ${\theta }^{2}$ = 0.0072, using the reduced set of 55 filtered SNPs, we found a 1 mph increase in walking pace was associated with a 65% (0.35, 0.23–0.53, *P* = 5.7 × 10^–7^) reduction in coronary artery disease risk.

The proportional hazards assumption held for all the Cox regression analyses (Supplementary Table 5).

### Two-sample Mendelian randomisation analysis

**Figure 1:**
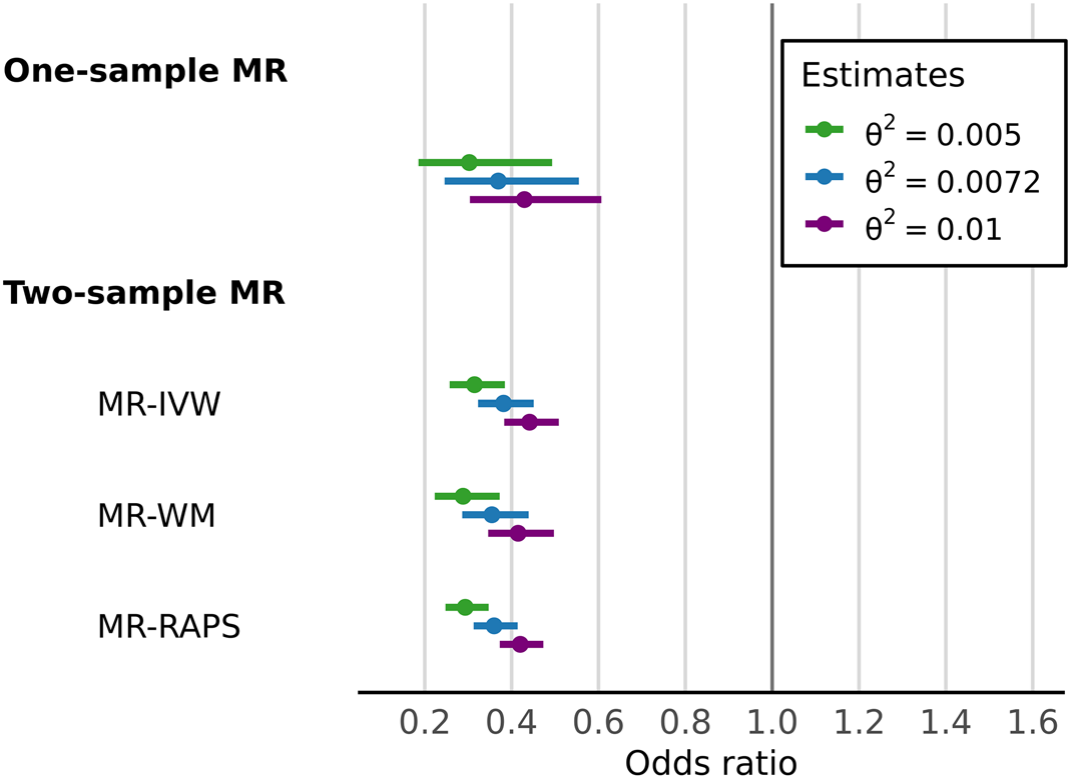
One-sample and two-sample Mendelian randomisation logistic regression analysis of self-reported walking pace on coronary artery disease. Effect of 1 mph increase in self-reported walking pace. The sensitivity parameter ${{\varvec{\theta}}}^{2}$ is the proportion of variance in walking pace explained by the genetic instruments on the latent scale, and was varied across a plausible range of values. IVW, inverse-variance weighted; WM, weighted-median; RAPS, robust adjusted profile score.

Table 3 shows the two-sample Mendelian randomisation results, which consistently supported a protective effect of walking pace on coronary artery disease. The one- and two-sample Mendelian randomisation provided causal effect estimates of very similar magnitude (Fig. 1).

**Table 3:** Two-sample Mendelian randomisation analysis of self-reported walking pace on coronary artery disease.

Method	θ^2^	Total effect		Direct effect (independent of BMI)		Indirect effect (mediated through BMI)		
		OR (95% CI)	*P* value	OR (95% CI)	*P* value	OR (95% CI)	*P* value	Proportion mediated (%)
MR-IVW	0.0050	0.31 (0.26–0.38)	1.6 × 10^–8^	0.53 (0.40–0.70)	0.025	0.59 (0.42–0.84)	3.0 × 10^–3^	45 (16–70)
MR-IVW	0.0072	0.38 (0.32–0.45)	1.6 × 10^–8^	0.59 (0.47–0.74)	0.025	0.65 (0.48–0.86)	3.0 × 10^–3^	45 (16–70)
MR-IVW	0.0100	0.44 (0.38–0.51)	1.6 × 10^–8^	0.64 (0.53–0.78)	0.025	0.69 (0.54–0.88)	3.0 × 10^–3^	45 (16–70)
MR-WM	0.0050	0.29 (0.22–0.37)	2.0 × 10^–6^	0.53 (0.37–0.76)	0.081	0.54 (0.35–0.84)	6.3 × 10^–3^	49 (15–77)
MR-WM	0.0072	0.35 (0.29–0.44)	2.0 × 10^–6^	0.59 (0.44–0.79)	0.081	0.60 (0.42–0.87)	6.3 × 10^–3^	49 (15–77)
MR-WM	0.0100	0.41 (0.35–0.50)	2.0 × 10^–6^	0.64 (0.50–0.82)	0.081	0.65 (0.48–0.88)	6.3 × 10^–3^	49 (15–77)
MR-RAPS	0.0050	0.29 (0.25–0.35)	9.7 × 10^–13^	0.49 (0.39–0.62)	2.8 × 10^–3^	0.60 (0.45–0.80)	4.8 × 10^–4^	42 (20–62)
MR-RAPS	0.0072	0.36 (0.31–0.41)	9.7 × 10^–13^	0.55 (0.46–0.67)	2.8 × 10^–3^	0.65 (0.51–0.83)	4.8 × 10^–4^	42 (20–62)
MR-RAPS	0.0100	0.42 (0.37–0.47)	9.7 × 10^–13^	0.60 (0.51–0.71)	2.8 × 10^–3^	0.69 (0.57–0.85)	4.8 × 10^–4^	42 (20–62)
MR-EGGER	0.0050	0.47 (0.20–1.09)	0.38	0.45 (0.20–1.02)	0.34	1.06 (0.33–3.42)	0.93	1 (− 740–480)
MR-EGGER	0.0072	0.54 (0.27–1.07)	0.38	0.51 (0.26–1.02)	0.34	1.04 (0.40–2.74)	0.93	1 (− 740–480)
MR-EGGER	0.0100	0.59 (0.33–1.06)	0.38	0.57 (0.32–1.01)	0.34	1.02 (0.44–2.36)	0.93	1 (− 740–480)

Effect per 1 mph increase in self-reported walking pace. IVW, inverse-variance weighted; WM, weighted-median; RAPS, robust adjusted profile score.

**Figure 2:**
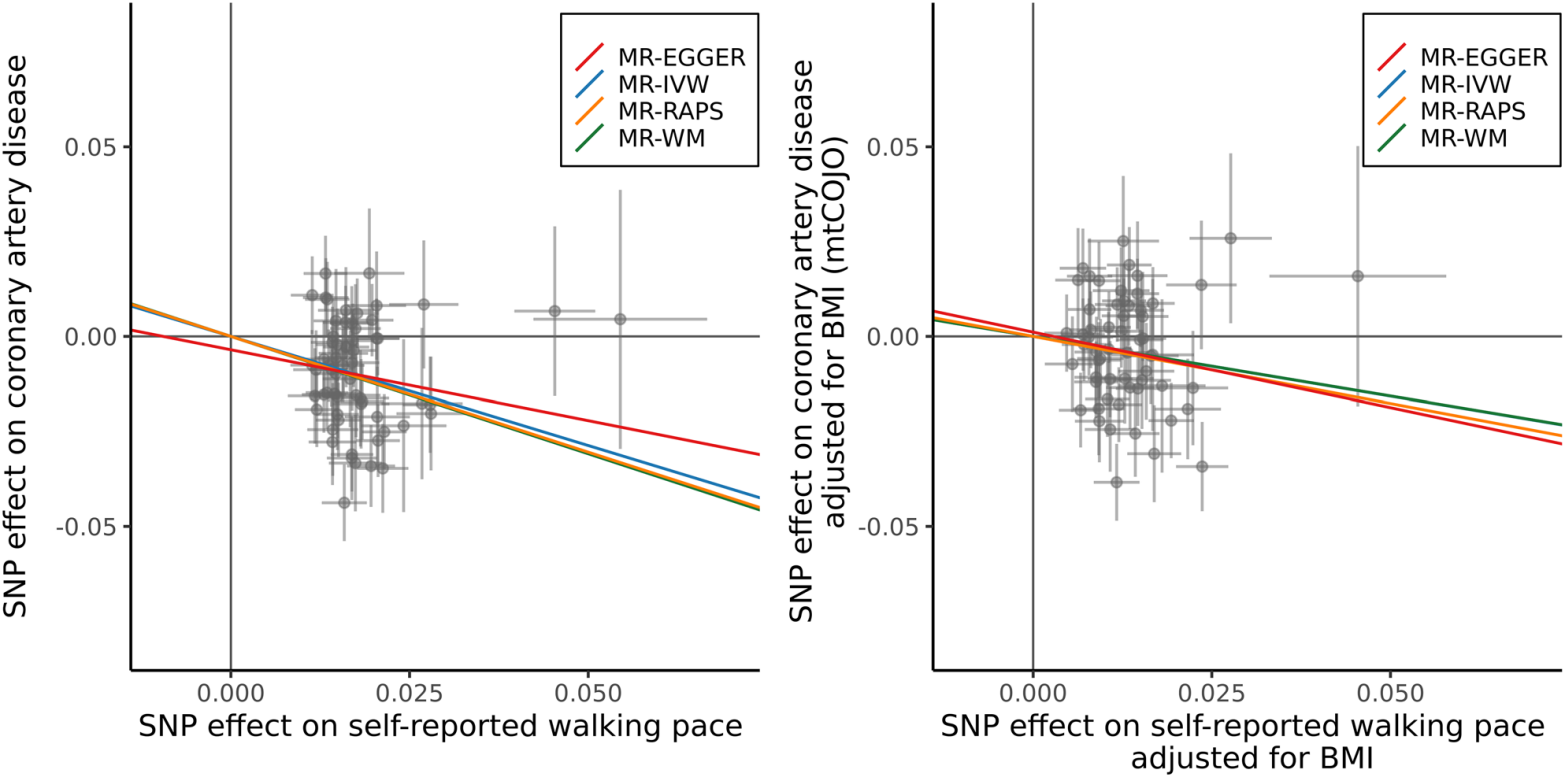
Scatter plot of individual SNP associations with self-reported walking pace and coronary artery disease. Associations presented before (left) and after (right) conditioning on BMI, with overlay of causal estimates from two-sample Mendelian randomisation analysis. IVW, inverse-variance weighted; WM, weighted-median; RAPS, robust adjusted profile score.

For ${\theta }^{2}$ = 0.0072, using the inverse-variance weighted method, a 1 mph increase in walking pace was associated with a 59% (odds ratio (OR) = 0.41, 95% CI 0.30–0.50, *P* = 1.6 × 10^–8^) reduction in coronary artery disease risk. Both the weighted median and MR-RAPS showed very similar effect sizes, which are compared in Fig. 2. Additionally, MR-Egger tests found no evidence of unbalanced horizontal pleiotropy (*P* > 0.44).

After using MR-Steiger filtering to remove 11 SNPs, the strength of association between walking pace and coronary artery disease remained largely unchanged (Supplementary Table 6). For ${\theta }^{2}$ = 0.0072, using the inverse-variance weighted method applied to a reduced set of 51 Steiger-filtered SNPs, a 1 mph increase in walking pace was associated with a 49% (OR = 0.51, 95% CI 0.37–0.69, *P* = 1.4 × 10^–5^) reduction in coronary artery disease risk. Weighted median and MR-RAPS sensitivity analyses showed broadly comparable effect sizes, and MR-Egger tests again found no evidence of unbalanced horizontal pleiotropy (*P* > 0.80).

### Mediation analysis on BMI

There was clear attenuation in the association of walking pace with coronary artery disease after conditioning for BMI, suggesting at least partial mediation through BMI. For ${\theta }^{2}$ = 0.0072, using the inverse-variance weighted method, compared to a total effect of a 1 mph increased walking pace reducing coronary artery disease risk by 62% (OR = 0.38, 95% CI 0.32–0.45, *P* = 1.6 × 10^–8^), the direct effect of walking pace independent of BMI was to reduce risk by 41% (OR = 0.59, 0.47–0.74, *P* = 0.025), and the proportion of the total effect mediated through BMI was 45% (95% CI 16–70%). Similar results for the direct effect and proportion mediated were found for the weighted median and MR-RAPS sensitivity analyses. Comparable findings were also found after performing Steiger filtering; for ${\theta }^{2}$ = 0.0072, compared to a total effect of 1 mph increased walking pace reducing coronary artery disease risk by 53% (OR = 0.47, 95% CI 0.40–0.55, *P* = 6.9 × 10^–6^), the direct effect of walking pace independent of BMI was to reduce risk by 36% (OR = 0.64, 95% CI 0.51–0.79, *P* = 0.043), corresponding to a proportion mediated through BMI of 40% (95% CI 6–70%).

## Discussion

Using genetic variants associated with walking pace, we performed one-sample and two-sample Mendelian randomisation to examine the potential causal role of walking pace on incident cardiovascular outcomes. We have aimed to identify the unconfounded association between walking pace and these outcomes to help understand the potential impact of interventions that encourage individuals to walk at a faster pace. This contrasts with previous studies that seek to use walking pace as an indicator of frailty or poor health status in order to identify those at increased cardiovascular risk^16,42^. All models considered, including a range of sensitivity analyses, were consistent with a causal effect of increased walking pace on reduced cardiovascular risk. We estimated that a 1 mph increase in self-reported walking pace consistently corresponded to roughly halving coronary artery disease risk across different methods and adjustments tested. While it is difficult to directly compare with previous per-category results, our estimates appeared stronger in magnitude than previous observational epidemiological studies^9–11^. We further added to previous studies by performing mediation analyses, which suggested that this causal effect was substantially mediated through BMI.

We also clarified findings from previous Mendelian randomisation studies on walking pace and cardiovascular disease risk by using a latent variable modelling approach that conceptualises self-reported categories of walking pace as coarsened approximations to an unobserved continuous measure of walking pace. This provides two advantages. First, the use of a latent exposure provides us with a more natural interpretation of effect sizes on a continuous scale, which in this case relate to the effect on cardiovascular outcomes of increases in walking pace in terms of miles per hour. Second, it avoids the need for additional strong assumptions about the underlying causal model. Without this approach, using categorical exposures in Mendelian randomisation assumes that there are no pleiotropic pathways from the genetic variant to outcome within levels of the categorical exposure. This is unlikely to be true, since altering the instrumental variable could plausibly modify continuous walking pace (and thereby possibly influence cardiovascular pathways and outcomes) without changing the discrete category of walking pace, creating such a pleiotropic pathway. We do, however, note that the magnitude of our effect sizes in terms of miles per hour were broadly consistent with the per-category effect sizes of previous Mendelian randomisation studies^15,18^, so our results suggest that these analyses were not overly influenced by such a bias.

We performed a range of sensitivity analyses to assess the instrumental variable assumptions underlying the Mendelian randomisation approach. After identifying and removing SNPs associated with related risk factors (BMI, cholesterol profile, blood pressure, socio-economic indices, and strenuous physical activity levels), the causal associations attenuated only slightly, suggesting our Mendelian randomisation findings were correctly orientated and robust to the effect of reverse causality. These results suggest that the causal associations could be primarily attributed to walking pace and were not capturing the effect of other related risk factors. Given the widespread pleiotropy observed for self-reported walking pace^18^, these were important sensitivity analyses that were not performed in previous Mendelian randomisation studies^15,18^.

Additionally, we found consistent effect size estimates across the one-sample and two-sample Mendelian randomisation approaches, which was a notable finding since these methods have different sources of bias. For instance, given the SNP-exposure effect sizes were estimated in the UK Biobank discovery sample, they were likely to be overestimated, which can cause winner’s curse bias. In the one-sample setting, this leads to bias towards the observational, confounded association, whereas in the two-sample setting, bias is towards the null. Thus, consistency of causal effect estimates across these methods suggests the impact of the winner’s curse was minimal.

Findings of a protective effect of walking pace on cardiovascular risk are supported by results from walking interventions^4^, which are informative about potential mechanisms. Brisk walking activity has been shown to have a beneficial effect on cardiometabolic risk factors and intermediaries such as aerobic capacity^43^, adiposity^44^, blood pressure^45^, haemostatic and inflammatory markers^46^, insulin sensitivity^47^ and endothelial function^48^. Additionally, studies have indicated brisk walking could have a direct effect on the cardiac system^49^, improving myocardial contraction and oxygen supply, and electric stability^50^. Further work on understanding mechanistic pathways could potentially involve using multivariable and mediation Mendelian randomisation approaches.

Our results help emphasise the role of walking in helping individuals meet the key guidelines for exercise, as brisk walking counts towards the recommended 150 min per week or more of moderate-intensity physical activity^51^. Moreover, our work suggests there are important benefits to be gained from walking at a faster pace, and this is consistent with recent studies that point to the intensity of physical activity being as important as the overall volume^52^.

We should also stress that our results do not detract from the existing epidemiological evidence for the health benefits of walking at a self-selected slower pace. We note that previous studies have observed that increased levels of other measures of walking activity, such as step count, duration, and frequency of walks, are associated with improvements in cardiovascular health^4^, particularly when compared to engaging in no walking activity. Indeed, it is also acknowledged that walking at a slower pace might be more feasible and accessible for certain population groups, such as those who are particularly inactive, overweight, frail or functionally compromised.

There are limitations to note. First, we were unable to validate the SNPs used for walking pace in an independent sample. Despite similar measures being available in some prospective cohorts^53,54^, we were unable to obtain the relevant data during the course of this study. Additionally, while we used external outcome data from CARDIoGRAMplusC4D as a form of validation, further work is needed to confirm the generalisability of our findings, particularly to non-European ancestry populations. We also note that the UK Biobank cohort is healthier than the wider population, which can lead to collider bias effects that distorts causal estimates^55^.

Second, it is acknowledged that these findings are dependent upon a subjective description of walking pace, and it is unclear how the causal effect sizes obtained here might compare with the true physiological effect from walking at or above a precise objectively measured speed. Despite this, studies have reported reasonable concordance between self-reported walking pace and objective assessments^56,57^, including when considering a comparable self-reported categorical walking pace variable with the measure in UK Biobank^58^. Validating our findings using accelerometer-derived physical activity metrics is also an important direction for future work^59^, though as yet there are still relatively few SNPs associated with device-measured metrics^60^ which means limited statistical power is currently available for performing such Mendelian randomisation analyses.

Third, we acknowledge that the latent variable approach requires strong untestable assumptions, which need to be carefully considered. Specifically, we have assumed that the latent exposure relates to clear-cut thresholds that define categories of walking pace, which are fixed and valid for all individuals. However, it is plausible that individuals may have different perceptions of what constitutes a “slow”, “steady/average” and “brisk” pace, since cognitive biases, personality traits and other factors could lead participants to over- or under-report their true category of walking pace. It could be more accurate instead to consider a model where random, individual-specific thresholds define categories of walking pace. This approach would assume an individual’s discrete category of walking pace depends on both their true latent continuous walking pace, in addition to a random effect that accounts for misclassification bias in their self-reported response. The tools to implement such an approach are currently lacking^19^.

Fourth, our approach assumes a linear relationship between walking pace and cardiovascular risk, though it is plausible that the true dose–response is curvilinear with risk reductions plateauing at the highest levels of walking pace^61^, consistent with other physical activity measures^62,63^.

Overall, our findings broadly support a protective causal effect of walking pace on cardiovascular disease risk, suggesting that interventions to promote more brisk walking could potentially lower the rate of cardiovascular outcomes at the population level.

### Supplementary Information

Supplementary Information.

## Data Availability

Analysis on UK Biobank data was performed under application number 33266, covered by the general ethical approval for UK Biobank studies from the National Health Service National Research Ethics. Because of the sensitive individual‐level nature of these data, they are not available to share by the authors but can be accessed by application directly to the UK Biobank. Publicly available genome‐wide association summary data from the CARDIoGRAMplusC4D consortium was used for validation analyses, and the details for accessing this data is available at the cited sources. Ethical approval and participant consent were obtained in each of the original studies that generated the data. Other datasets from this study are available from the corresponding author.
