# Supplementary material for: Mendelian randomisation and mediation analysis of self-reported walking pace and coronary artery disease
Source: Sci Rep. 2024 May 1;14:9995. doi: 10.1038/s41598-024-60398-8 (PMC11063179; doi:10.1038/s41598-024-60398-8)
Supplement: Supplementary file 1 — Supplementary Information. [file 41598_2024_60398_MOESM1_ESM.pdf]

Supplementary Information: Mendelian randomisation and mediation analysis of self-reported walking pace and coronary artery disease

Iain R. Timmins, Francesco Zaccardi, Thomas Yates, and Frank Dudbridge

Supplementary Figures

Supplementary Figure 1: Flow chart for exclusions made in UK Biobank for resultant analytical sample. 2

Supplementary Tables

Supplementary Table 1: Genetic instruments for self-reported walking pace. Effect sizes/s.e. from ordinal probit regression, with R^2 (latent scale) and F-statistics. 3
Supplementary Table 2: Participant characteristics of analytical sample at baseline and by genetically predicted self-reported walking pace, by deciles. 5
Supplementary Table 3: One-sample Mendelian randomisation logistic regression analysis of self-reported walking pace on coronary artery disease in UK Biobank. Effect of 1 mph increase in self-reported walking pace. 6
Supplementary Table 4: MR-Steiger directionality test for genetic instruments for self-reported walking pace. For each potential confounder/outcome, Steiger's Z-test is used to infer the direction of causality. 7
Supplementary Table 5: Statistical test of the proportional hazards assumption. 10
Supplementary Table 6: Two-sample Mendelian randomisation analysis of self-reported walking pace on coronary artery disease, with Steiger filtering of SNPs. Effect per 1 mph increase in self-reported walking pace. 11

Supplementary Figure 1: Flow chart for exclusions made in UK Biobank for resultant analytical sample.

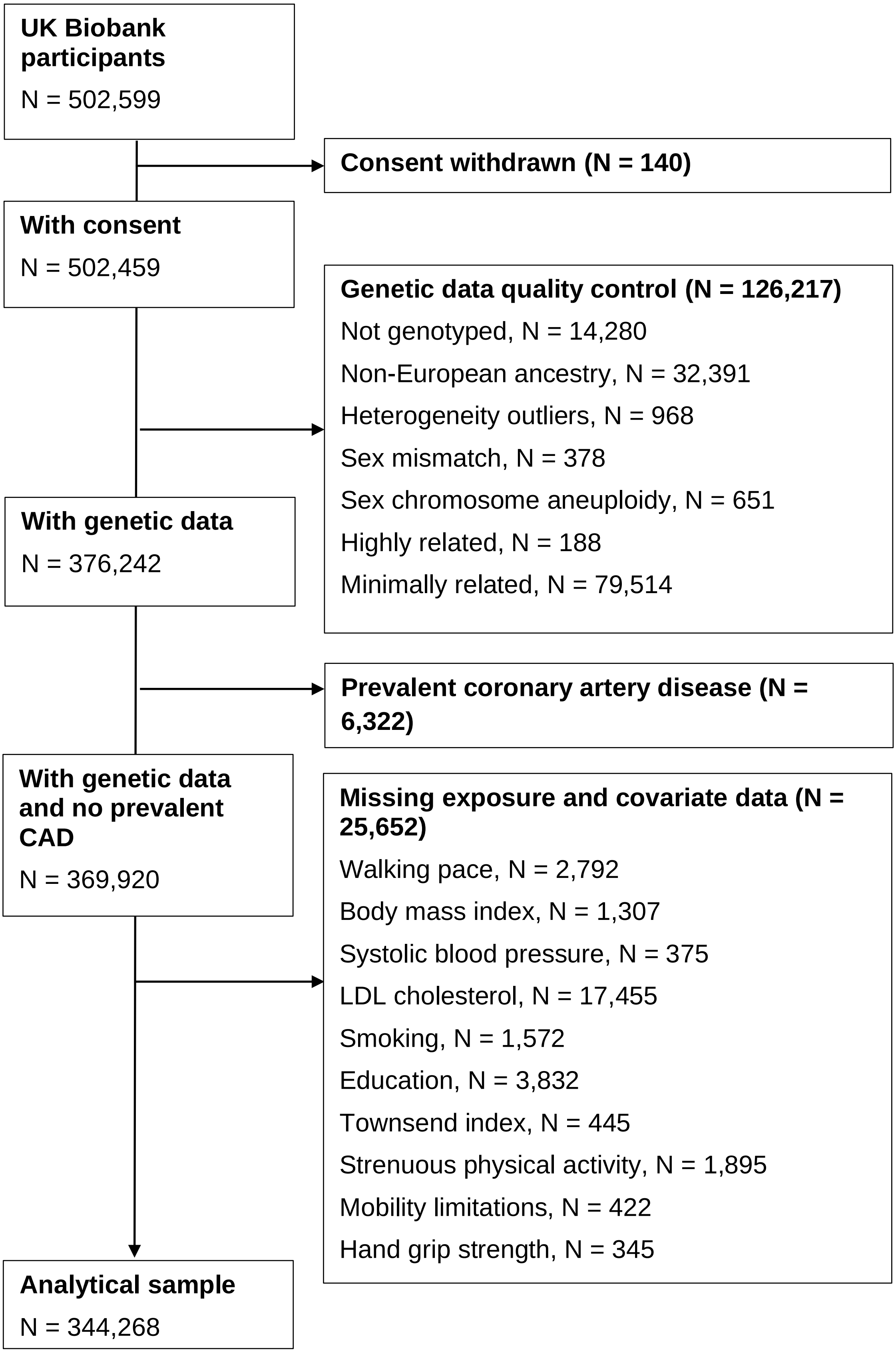

Supplementary Table 1: Genetic instruments for self-reported walking pace. Effect sizes/s.e. from ordinal probit regression, with R^2 (latent scale) and F-statistics. maf, minor allele frequency.

SNP	CHR	BP	A1	A2	maf	Beta	s.e.	R^2-latent scale	F-statistic
rs12735861	1	1,562,895	C	T	0.41	0.013	0.003	$7.7x10^{-5}$	17.6
rs2297600	1	32,207,581	T	G	0.17	0.018	0.004	$9.8x10^{-5}$	22.4
rs113825410	1	40,057,543	A	G	0.22	0.014	0.004	$5.0x10^{-5}$	11.4
rs699784	1	117,200,739	T	C	0.23	-0.017	0.004	$7.4x10^{-5}$	17.0
rs10158328	1	155,027,763	A	G	0.46	0.011	0.003	$5.9x10^{-5}$	13.4
rs11548200	1	156,290,656	T	C	0.07	0.024	0.006	$8.2x10^{-5}$	18.7
rs6674490	1	185,136,458	C	A	0.41	-0.011	0.003	$5.1x10^{-5}$	11.7
rs12127169	1	243,615,023	C	T	0.11	-0.028	0.005	$1.5x10^{-5}$	34.1
rs1531133	2	46,843,631	A	G	0.42	-0.014	0.003	$9.8x10^{-5}$	22.3
rs13005495	2	60,157,097	T	G	0.42	0.015	0.003	$9.7x10^{-5}$	22.1
rs55680124	2	105,984,624	C	T	0.15	0.020	0.004	$1.2x10^{-4}$	27.9
rs17698630	2	135,691,725	A	G	0.18	-0.016	0.004	$1.2x10^{-4}$	27.2
rs13409533	2	144,139,538	G	T	0.19	-0.017	0.004	$6.7x10^{-5}$	15.2
rs2054079	2	226,486,752	C	T	0.32	-0.012	0.003	$7.2x10^{-5}$	16.5
rs62246314	3	9,504,099	G	A	0.10	0.019	0.005	$6.4x10^{-5}$	14.6
rs2920503	3	12,324,230	C	T	0.28	-0.013	0.003	$6.5x10^{-5}$	14.7
rs2280406	3	49,941,436	G	A	0.49	0.017	0.003	$1.5x10^{-4}$	33.8
rs62253608	3	52,989,383	C	T	0.34	-0.016	0.003	$1.0x10^{-4}$	23.7
rs830627	3	71,675,270	G	A	0.42	-0.016	0.003	$1.2x10^{-4}$	27.4
rs6763292	3	129,044,705	A	G	0.22	-0.021	0.004	$1.6x10^{-4}$	36.1
rs9844666	3	135,974,216	G	A	0.24	0.017	0.003	$1.1x10^{-4}$	25.0
rs798748	4	1,716,770	T	C	0.38	0.013	0.003	$6.6x10^{-5}$	15.1
rs362307	4	3,241,845	C	T	0.07	0.027	0.006	$8.6x10^{-5}$	19.6
rs72636700	4	68,019,509	T	C	0.17	0.018	0.004	$5.6x10^{-5}$	12.9
rs13107325	4	103,188,709	C	T	0.07	0.045	0.006	$3.1x10^{-4}$	70.7
rs115202226	4	133,802,757	A	G	0.00	-0.102	0.021	$1.1x10^{-4}$	24.5
rs57800857	4	140,863,365	A	C	0.36	-0.013	0.003	$7.4x10^{-5}$	16.9
rs4134943	6	20,483,407	C	T	0.20	-0.017	0.004	$1.0x10^{-4}$	23.7
rs9366651	6	26,336,696	G	T	0.49	-0.020	0.003	$2.2x10^{-4}$	50.4
rs1061801	6	33,282,338	G	A	0.19	0.012	0.004	$4.7x10^{-5}$	10.8
rs205262	6	34,563,164	A	G	0.27	0.020	0.003	$1.4x10^{-4}$	31.6
rs4715208	6	50,829,471	A	G	0.25	0.016	0.003	$1.1x10^{-4}$	25.1
rs4839898	6	97,546,759	G	A	0.10	-0.027	0.005	$1.2x10^{-4}$	26.7
rs7804774	7	66,903,028	A	G	0.19	-0.017	0.004	$7.8x10^{-5}$	17.9
rs10452738	7	69,453,714	A	G	0.32	0.015	0.003	$9.5x10^{-5}$	21.6
rs34982641	7	75,135,803	G	A	0.29	-0.019	0.003	$1.4x10^{-4}$	32.5
rs3857834	7	113,541,103	C	T	0.38	-0.017	0.003	$1.4x10^{-4}$	31.5
rs145401744	8	143,495,754	T	C	0.01	-0.054	0.012	$8.8x10^{-5}$	20.1

rs10828258	10	21,929,734	A	G	0.32	0.020	0.003	$1.5x10^{-4}$	34.3
rs7924036	10	65,191,645	G	T	0.50	-0.018	0.003	$1.9x10^{-4}$	43.3
rs2439823	10	99,778,226	A	G	0.45	0.015	0.003	$8.9x10^{-5}$	20.3
rs10883618	10	103,117,653	G	A	0.37	-0.013	0.003	$9.1x10^{-5}$	20.9
rs4109292	10	126,710,654	G	A	0.50	-0.013	0.003	$8.8x10^{-5}$	20.2
rs11039324	11	47,665,686	G	A	0.40	0.021	0.003	$2.2x10^{-4}$	51.2
rs10750025	11	113,424,042	C	T	0.32	0.016	0.003	$1.1x10^{-4}$	25.8
rs10862220	12	81,430,599	T	G	0.32	-0.016	0.003	$1.2x10^{-4}$	27.9
rs6539771	12	84,077,443	C	T	0.36	0.021	0.003	$1.7x10^{-4}$	38.8
rs61954972	12	123,074,167	T	G	0.26	0.018	0.003	$9.0x10^{-5}$	20.5
rs12883788	14	33,303,540	C	T	0.46	0.015	0.003	$1.3x10^{-4}$	28.5
rs8010773	14	46,956,863	T	C	0.38	0.014	0.003	$1.1x10^{-4}$	24.9
rs8011870	14	80,173,397	G	A	0.29	0.015	0.003	$1.2x10^{-4}$	27.5
rs78551446	14	100,993,256	T	C	0.22	0.020	0.004	$1.3x10^{-4}$	28.7
rs7187776	16	28,857,645	A	G	0.40	0.018	0.003	$1.5x10^{-4}$	33.2
rs34898535	16	31,025,641	C	T	0.38	-0.015	0.003	$1.2x10^{-4}$	27.4
rs9972653	16	53,814,363	G	T	0.40	0.021	0.003	$1.6x10^{-4}$	37.0
rs1652376	18	21,109,466	G	T	0.46	-0.014	0.003	$6.6x10^{-5}$	15.0
rs2469878	18	38,240,381	C	T	0.33	0.017	0.003	$1.4x10^{-4}$	32.0
rs784257	18	53,397,199	T	C	0.18	0.028	0.004	$2.5x10^{-4}$	58.2
rs67625472	19	4,968,620	T	C	0.28	0.014	0.003	$7.4x10^{-5}$	17.0
rs273512	19	18,224,729	C	T	0.40	0.017	0.003	$1.6x10^{-4}$	36.1
rs11881199	19	18,837,459	T	C	0.50	-0.014	0.003	$1.2x10^{-4}$	27.5
rs12461902	19	30,265,235	G	A	0.33	0.013	0.003	$8.7x10^{-5}$	19.9
rs1667369	19	37,489,617	A	C	0.37	0.014	0.003	$8.9x10^{-5}$	20.4
rs35741895	19	47,982,462	A	G	0.12	0.020	0.005	$1.1x10^{-4}$	24.4
rs143384	20	34,025,756	A	G	0.40	-0.014	0.003	$1.2x10^{-4}$	27.4

Supplementary Table 2: Participant characteristics of analytical sample at baseline and by genetically predicted self-reported walking pace, by deciles.

	65 SNP Genetic predictor of walking pace			
**Variable**	**Bottom Decile**	**Deciles 2-9**	**Top Decile**	**Total Sample**
Participants	34,427 (10.0%)	275,415 (80.0%)	34,426 (10.0%)	344,268
Female gender	18,657 (54.2%)	150,408 (54.6%)	18,798 (54.6%)	187,863 (54.6%)
Age (years)	57.0 (8.0)	57.1 (8.0)	57.1 (8.0)	57.1 (8.0)
Body mass index (kg/m^2)	28.0 (5.0)	27.3 (4.7)	26.7 (4.4)	27.3 (4.7)
Systolic blood pressure (mmHg)	138.3 (18.6)	138.0 (18.6)	137.5 (18.7)	138.0 (18.6)
LDL cholesterol (mmol/l)	3.6 (0.9)	3.6 (0.9)	3.6 (0.9)	3.6 (0.9)
Smoking status				
Never	18,366 (53.3%)	149,992 (54.5%)	19,138 (55.6%)	187,496 (54.5%)
Former	12,206 (35.5%)	97,112 (35.3%)	11,996 (34.8%)	121,314 (35.2%)
Current	3,855 (11.2%)	28,311 (10.3%)	3,292 (9.6%)	35,458 (10.3%)
Years of education	14.7 (5.1)	15.0 (5.1)	15.4 (5.0)	15.0 (5.1)
Townsend deprivation index	-1.4 (3.0)	-1.5 (3.0)	-1.6 (2.9)	-1.5 (3.0)
History of diabetes	1,752 (5.1%)	11,631 (4.2%)	1,261 (3.7%)	14,644 (4.3%)
Participate in strenuous physical activity	9,006 (26.2%)	76,451 (27.8%)	10,168 (29.5%)	95,625 (27.8%)
Health status (classified as healthy)	22,133 (64.3%)	181,947 (66.1%)	23,102 (67.1%)	227,182 (66.0%)
Mobility limitation	14,575 (42.3%)	109,263 (39.7%)	13,014 (37.8%)	136,852 (39.8%)
Grip strength (kg)	30.6 (11.1)	30.9 (11.0)	31.3 (11.0)	30.9 (11.0)
Cardiorespiratory fitness* (mL·kg^{-1}·min^{-1})	35.6 (10.3)	36.2 (10.3)	36.6 (10.4)	36.2 (10.3)
Coronary artery disease events	1,090 (3.2%)	7,979 (2.9%)	924 (2.7%)	9,993 (2.9%)

Supplementary Table 3: One-sample Mendelian randomisation logistic regression analysis of self-reported walking pace on coronary artery disease in UK Biobank. Effect of 1 mph increase in self-reported walking pace.

SNPs	θ^2	Odds ratio (95% CI) per 1 mph increase in walking pace	P-value
66	0.0050	0.30 (0.19-0.49)	$1.6x10^{-6}$
66	0.0072	0.37 (0.25-0.55)	$1.6x10^{-6}$
66	0.0100	0.43 (0.30-0.61)	$1.6x10^{-6}$
55 (Steiger-filtered)	0.0050	0.29 (0.17-0.47)	$1.1x10^{-6}$
55 (Steiger-filtered)	0.0072	0.35 (0.23-0.54)	$1.1x10^{-6}$
55 (Steiger-filtered)	0.0100	0.41 (0.29-0.59)	$1.1x10^{-6}$

Supplementary Table 4: MR-Steiger directionality test for genetic instruments for self-reported walking pace. For each potential confounder/outcome, Steiger's Z-test is used to infer the direction of causality.

D denotes to the causal direction: (+) is interpreted such that the causal direction is walking pace → confounder/outcome, and (-) is interpreted such that confounder/outcome → walking pace.

SNP	Confounder/Outcome																							
	Body mass index		Systolic blood pressure		LDL cholesterol		Smoking status		Educational attainment		History of diabetes		Strenuous physical activity		Health status		Mobility limitation		Hand grip strength		Cardio-respiratory fitness		Coronary artery disease	
	D	P-val	D	P-val	D	P-val	D	P-val	D	P-val	D	P-val	D	P-val	D	P-val	D	P-val	D	P-val	D	P-val	D	P-val
rs2297600	-	0.76	+	0.08	+	0.04	+	0.95	+	0.96	+	0.23	+	0.11	+	0.15	+	0.05	+	0.02	+	0.24	+	0.18
rs113825410	-	0.42	+	0.06	+	0.16	+	0.04	+	0.11	-	0.53	+	0.20	+	0.67	+	0.73	+	0.02	+	0.71	+	0.30
rs699784	+	0.13	+	0.06	+	0.02	+	0.20	+	0.02	+	0.01	+	0.06	+	0.02	+	0.02	+	0.02	+	0.44	+	0.09
rs10158328	+	0.60	+	0.08	+	0.18	+	0.22	+	0.41	+	0.07	+	0.21	+	0.19	+	0.23	+	0.38	+	0.40	+	0.08
rs11548200	+	0.93	+	0.17	+	0.06	+	0.18	+	0.71	+	0.10	+	0.98	+	0.03	+	0.11	+	0.10	+	0.11	+	0.05
rs6674490	+	0.72	+	0.17	+	0.15	+	0.07	+	0.78	+	0.13	+	0.40	+	0.06	+	0.07	+	0.82	-	0.93	+	0.06
rs12127169	+	0.47	+	0.03	+	$4.2x10^{-3}$	+	$2.6x10^{-3}$	+	0.04	+	0.02	+	0.03	+	$1.9x10^{-3}$	+	0.11	+	0.02	+	0.99	+	0.01
rs1531133	+	0.94	+	0.05	+	0.13	+	0.06	+	0.01	+	0.08	+	0.03	+	0.10	+	$4.0x10^{-3}$	+	0.18	+	0.76	+	0.04
rs13005495	+	0.34	+	0.03	+	0.02	+	0.70	+	0.34	+	0.04	+	0.02	+	0.03	+	0.05	+	0.09	-	0.79	+	0.01
rs55680124	-	0.81	+	0.12	+	0.01	+	0.72	+	0.09	+	0.49	+	0.40	+	0.06	+	0.02	+	0.14	-	0.94	+	0.03
rs17698630	+	0.85	-	0.70	+	0.52	+	0.25	+	0.06	+	0.14	+	0.14	+	0.06	+	0.06	-	0.16	-	0.90	+	0.28
rs13409533	+	0.04	+	0.04	+	0.04	+	0.11	+	0.05	+	0.02	+	0.02	+	0.03	+	0.08	+	0.07	+	0.40	+	0.15
rs2054079	+	0.63	+	0.77	+	0.07	+	0.17	-	0.95	+	0.07	+	0.10	+	0.23	+	0.17	+	0.36	-	0.83	+	0.09
rs62246314	-	0.49	+	0.16	+	0.06	+	0.28	+	0.09	+	0.08	+	0.06	+	0.08	+	0.02	+	0.05	-	0.96	+	0.42
rs2920503	-	0.26	+	0.05	+	0.59	+	0.05	+	0.27	+	0.37	+	0.05	+	0.09	+	0.03	+	0.30	+	0.29	+	0.06
rs2280406	-	$1.3x10^{-5}$	+	0.58	+	0.01	+	0.19	-	$4.1x10^{-3}$	+	0.33	+	0.19	+	$3.0x10^{-3}$	+	0.07	+	0.04	+	0.81	+	0.05
rs62253608	-	0.99	+	0.38	+	0.05	+	0.06	+	0.86	+	0.06	+	0.07	+	0.01	+	$4.0x10^{-3}$	-	0.89	+	0.42	+	0.01
rs830627	+	0.23	+	0.09	+	0.09	+	0.58	+	0.67	+	0.15	+	0.01	+	0.04	+	0.02	+	0.55	+	0.70	+	0.03

rs6763292	+	2.3×10^{-4}	+	0.02	+	2.9×10^{-3}	+	4.0×10^{-3}	+	8.9×10^{-4}	+	0.01	+	4.1×10^{-3}	+	0.05	+	0.08	+	0.11	+	0.98	+	0.01
rs9844666	-	0.28	+	0.09	+	0.63	+	0.04	+	0.01	+	0.13	+	0.06	+	7.5×10^{-4}	+	0.06	+	0.28	+	0.55	+	0.06
rs798748	+	0.01	+	0.03	+	0.41	+	0.03	+	0.13	+	0.02	+	0.14	+	0.01	+	0.34	+	0.22	+	0.30	+	0.03
rs362307	-	0.23	+	0.29	+	0.44	+	0.01	+	0.84	+	0.19	+	0.24	+	0.01	+	0.03	+	0.05	-	0.82	+	0.05
rs72636700	+	0.32	+	0.06	+	0.11	+	0.27	-	0.85	+	0.01	+	0.09	+	0.72	+	0.91	+	0.21	-	0.78	+	0.09
rs13107325	-	0.02	+	0.44	+	0.14	+	0.01	+	0.07	+	0.01	+	0.02	+	0.09	+	0.10	+	0.01	+	0.44	+	5.2×10^{-5}
rs57800857	-	0.14	+	0.16	+	0.06	+	0.75	+	0.40	+	0.48	+	0.54	+	3.6×10^{-3}	+	0.01	+	0.86	+	0.96	+	0.05
rs4134943	+	0.02	+	0.34	+	0.02	+	0.03	+	0.01	+	0.13	+	0.01	+	0.41	+	0.24	+	0.29	+	0.62	+	0.09
rs9366651	+	0.10	+	8.6×10^{-4}	+	0.12	+	0.01	-	0.84	+	0.06	+	0.59	+	0.12	+	0.15	+	0.36	+	0.69	+	3.8×10^{-3}
rs1061801	-	0.06	+	0.33	+	0.13	-	0.92	-	0.17	+	0.65	+	0.59	+	0.03	+	0.01	+	6.5×10^{-4}	+	0.61	+	0.69
rs205262	-	1.0×10^{-3}	+	0.02	+	0.03	+	0.05	+	0.01	+	2.2×10^{-3}	+	0.01	+	0.95	+	0.44	+	0.15	-	0.38	+	0.03
rs4715208	-	6.0×10^{-5}	+	0.06	+	0.14	+	0.02	+	0.14	+	0.37	+	0.03	+	0.01	+	0.02	+	3.3×10^{-3}	+	0.43	+	0.09
rs4839898	+	0.10	+	0.02	+	0.01	+	0.01	+	0.01	+	0.02	+	0.20	+	0.02	+	0.14	+	0.08	+	0.88	+	0.01
rs7804774	+	0.03	+	0.05	+	0.05	+	0.21	+	0.11	+	0.01	+	0.01	+	0.01	+	0.01	+	1.9×10^{-3}	+	0.27	+	0.04
rs10452738	+	0.51	+	0.88	+	0.08	+	0.31	+	0.01	+	0.06	+	0.03	+	0.02	+	0.04	+	0.03	+	0.39	+	0.17
rs236660	-	1.5×10^{-3}	-	0.70	+	0.25	+	0.18	+	0.55	+	0.09	+	0.01	+	0.01	+	0.02	+	0.16	+	0.37	+	0.08
rs3857834	-	0.65	+	0.03	+	0.13	+	0.01	+	0.03	+	0.01	+	0.03	+	0.08	+	0.10	+	0.01	-	0.92	+	0.02
rs145401744	+	0.34	+	0.02	+	0.03	+	0.33	+	0.54	+	0.05	+	0.02	+	0.06	+	0.16	+	0.13	+	0.24	+	0.04
rs10828258	-	0.29	+	0.12	+	0.05	+	0.11	+	0.22	+	1.9×10^{-3}	+	0.05	+	0.12	+	0.10	+	0.01	-	0.97	+	4.2×10^{-3}
rs7924036	+	0.26	+	0.18	+	0.59	+	0.01	-	0.82	+	0.11	+	0.02	+	1.9×10^{-3}	+	1.9×10^{-4}	+	0.09	+	0.78	+	0.01
rs2439823	-	0.11	+	0.02	+	0.15	+	0.15	+	0.04	+	0.01	+	0.23	+	0.01	+	2.6×10^{-4}	+	0.01	+	0.32	+	0.11
rs10883618	+	0.43	+	0.24	+	0.04	+	0.26	+	0.17	+	0.57	+	0.06	+	0.01	+	4.6×10^{-3}	+	0.43	+	0.80	+	0.04
rs4109292	-	0.42	+	0.05	+	0.03	+	0.19	+	0.15	+	0.08	+	0.91	+	0.03	+	0.06	+	0.76	+	0.65	+	0.03
rs11039324	-	0.09	+	0.01	+	0.01	+	0.06	+	1.4×10^{-4}	+	0.02	+	4.3×10^{-4}	+	0.02	+	0.08	+	0.14	-	0.89	+	4.8×10^{-4}
rs10750025	+	0.04	+	0.01	+	0.26	+	0.01	+	0.01	+	0.03	+	0.01	+	1.1×10^{-4}	+	3.9×10^{-3}	+	0.65	+	0.79	+	0.16
rs10862220	+	0.04	+	0.01	+	0.16	+	0.02	+	0.09	+	0.01	+	0.01	+	0.06	+	0.18	+	0.19	+	0.03	+	0.10
rs6539771	+	0.01	+	0.01	+	1.6×10^{-3}	+	0.13	+	0.45	+	3.5×10^{-3}	+	0.01	+	0.02	+	0.07	+	0.01	+	0.64	+	4.4×10^{-3}

rs61954972	-	0.12	+	0.03	+	0.14	+	0.01	+	0.71	+	0.01	+	0.04	+	4.5×10^{-3}	+	1.4×10^{-3}	+	4.8×10^{-3}	+	0.21	+	0.02
rs12883788	-	0.04	+	0.11	+	0.11	+	0.19	+	0.57	+	0.05	+	0.04	+	0.03	+	0.02	+	0.04	+	0.48	+	0.29
rs8010773	+	0.91	+	0.06	+	0.04	+	0.61	+	0.20	+	0.07	+	0.06	+	0.03	+	0.19	+	0.22	-	0.32	+	0.24
rs8011870	+	0.09	+	0.03	+	0.04	+	0.04	+	0.01	+	0.03	+	0.01	+	0.08	+	0.14	+	0.05	+	0.30	+	0.16
rs7187776	-	1.6×10^{-3}	+	0.05	+	0.01	+	2.2×10^{-3}	-	0.43	+	0.02	+	3.5×10^{-3}	+	0.05	+	0.01	+	0.15	+	0.11	+	0.01
rs34898535	-	1.8×10^{-5}	+	0.53	+	0.22	+	0.29	+	0.31	+	0.17	+	0.22	+	7.2×10^{-4}	+	1.4×10^{-3}	+	0.37	+	0.78	+	0.11
rs9972653	-	1.1×10^{-56}	+	0.14	+	0.33	+	0.01	+	1.5×10^{-4}	-	0.24	+	0.04	+	0.05	+	0.21	+	0.93	+	0.43	+	8.2×10^{-4}
rs1652376	-	0.02	+	0.06	+	0.14	+	0.05	-	0.78	+	0.06	+	0.71	+	0.08	+	0.04	+	5.6×10^{-4}	+	0.97	+	0.06
rs2469878	+	0.05	+	0.02	+	0.12	+	0.03	+	0.03	+	0.01	+	0.01	+	0.03	+	0.17	+	0.13	+	0.98	+	0.01
rs784257	+	0.14	+	0.01	+	8.7×10^{-4}	+	3.5×10^{-3}	+	0.34	+	4.0×10^{-4}	+	4.2×10^{-4}	+	0.01	+	0.10	+	0.14	+	0.08	+	2.5×10^{-4}
rs67625472	+	0.01	+	0.93	+	0.07	+	0.23	+	0.36	+	0.13	+	0.12	+	1.0×10^{-3}	+	2.4×10^{-3}	+	0.12	+	0.05	+	0.03
rs273512	+	0.96	+	0.01	+	0.18	+	0.01	+	0.38	+	0.05	+	0.01	+	0.10	+	0.12	+	0.07	-	0.82	+	4.8×10^{-3}
rs11881199	-	0.05	+	0.03	+	0.03	+	0.02	+	0.05	+	0.26	+	0.01	+	0.01	+	0.01	+	0.04	+	0.23	+	0.02
rs12461902	-	0.06	+	0.54	+	0.06	+	0.03	+	0.05	+	0.07	+	0.32	+	0.01	+	0.05	+	0.04	+	0.99	+	0.44
rs9676450	+	0.31	+	0.14	+	0.31	+	0.13	+	0.07	+	0.08	+	0.08	+	0.10	+	0.30	-	0.90	-	0.72	+	0.05
rs35741895	+	1.6×10^{-3}	+	0.04	+	0.04	+	0.06	+	0.01	+	0.18	+	0.02	+	0.02	+	0.04	-	0.64	+	1.00	+	0.05
rs143384	+	3.9×10^{-3}	+	0.75	+	0.61	+	0.08	+	0.91	+	0.01	+	0.11	+	0.01	+	0.01	+	0.04	+	0.56	+	0.02

Supplementary Table 5: Statistical test of the proportional hazards assumption.

Model	Correlation coefficient*	P-value
Mendelian randomisation – basic adjustment	0.02	0.16
Mendelian randomisation – further adjusted for BMI	0.01	0.20

*The Pearson's correlation coefficient and P-values between the scaled Schoenfeld residuals for walking pace in the Cox regression and rank-normalised natural logarithm of follow-up time.

Supplementary Table 6: Two-sample Mendelian randomisation analysis of self-reported walking pace on coronary artery disease, with Steiger filtering of SNPs. Effect per 1 mph increase in self-reported walking pace.

		Total effect		Direct effect (independent of BMI)		Indirect effect (mediated through BMI)		
**Method**	**θ^2**	**OR (95% CI)**	**P-value**	**OR (95% CI)**	**P-value**	**OR (95% CI)**	**P-value**	**Proportion mediated (%)**
MR-IVW	0.0050	0.40 (0.33-0.49)	6.90×10^{-6}	0.58 (0.45-0.76)	0.043	0.70 (0.50-0.96)	0.029	40 (6-70)
MR-IVW	0.0072	0.47 (0.40-0.55)	6.90×10^{-6}	0.64 (0.51-0.79)	0.043	0.74 (0.56-0.97)	0.029	40 (6-70)
MR-IVW	0.0100	0.53 (0.46-0.61)	6.90×10^{-6}	0.68 (0.57-0.82)	0.043	0.77 (0.61-0.97)	0.029	40 (6-70)
MR-WM	0.0050	0.43 (0.33-0.54)	6.50×10^{-4}	0.57 (0.41-0.79)	0.091	0.75 (0.49-1.13)	0.16	34 (-16-73)
MR-WM	0.0072	0.49 (0.40-0.60)	6.50×10^{-4}	0.63 (0.48-0.82)	0.091	0.79 (0.56-1.10)	0.16	34 (-16-73)
MR-WM	0.0100	0.55 (0.46-0.65)	6.50×10^{-4}	0.67 (0.53-0.85)	0.091	0.81 (0.61-1.08)	0.16	34 (-16-73)
MR-RAPS	0.0050	0.38 (0.32-0.45)	1.50×10^{-8}	0.55 (0.44-0.68)	5.8×10^{-3}	0.70 (0.53-0.91)	9.4×10^{-3}	37 (10-60)
MR-RAPS	0.0072	0.45 (0.39-0.52)	1.50×10^{-8}	0.60 (0.51-0.72)	5.8×10^{-3}	0.74 (0.59-0.93)	9.4×10^{-3}	37 (10-60)
MR-RAPS	0.0100	0.51 (0.45-0.57)	1.50×10^{-8}	0.65 (0.56-0.76)	5.8×10^{-3}	0.78 (0.64-0.94)	9.4×10^{-3}	37 (10-60)
MR-EGGER	0.0050	0.50 (0.21-1.18)	0.43	0.29 (0.12-0.69)	0.16	1.74 (0.51-5.94)	0.38	-64 (-1550-1010)
MR-EGGER	0.0072	0.56 (0.27-1.15)	0.43	0.35 (0.17-0.73)	0.16	1.59 (0.57-4.45)	0.38	-64 (-1550-1010)
MR-EGGER	0.0100	0.61 (0.33-1.13)	0.43	0.41 (0.22-0.77)	0.16	1.48 (0.62-3.52)	0.38	-64 (-1550-1010)
